# The miR-17-92 cluster/QKI2/β-catenin axis promotes osteosarcoma progression

**DOI:** 10.18632/oncotarget.23935

**Published:** 2018-01-05

**Authors:** Hongbo Yang, Zhibin Peng, Min Liang, Yubo Zhang, Yangyang Wang, Tianwen Huang, Yudong Jiang, Bo Jiang, Yansong Wang

**Affiliations:** ^1^ Department of Orthopaedic Surgery, The First Affiliated Hospital of Harbin Medical University, Harbin, China

**Keywords:** osteosarcoma, miR-17-92 cluster, QKI2, β-catenin, progression

## Abstract

Quaking(QKI) is an RNA binding protein, and it has been shown to serve as a tumor suppressor. However, the expression and functions of QKI in osteosarcoma progression remain poorly understood. In this study, we aimed to explore the expression of QKI2 in osteosarcoma tissues and to determine the mechanisms underlying aberrant QKI2 expression and the effect of QKI2 on osteosarcoma progression. We found that QKI2 was significantly down-regulated in osteosarcoma tissues compared with adjacent normal bone tissues. Using a series of molecular biological techniques, we demonstrated that all members of the miR-17-92 cluster were up-regulated and contributed to the down-regulation of QKI2 expression in osteosarcoma. Functional examination showed that QKI2 inhibited the proliferation, migration and invasion of osteosarcoma cells via decreasing the expression of β-catenin. Conclusively, we revealed that the regulatory axis consisting of the miR-17-92 cluster/QKI2/β-catenin plays a crucial role in the development and progression of osteosarcoma.

## INTRODUCTION

Osteosarcoma(OS) is the most common primary malignant, aggressive neoplasm of the bone and has a predilection for occurring in children and adolescents [1]. The metaphyses of long bones are one of the most common sites for osteosarcoma; approximately 50% of cases are localized in the distal femoral and proximal tibial knee region, and approximately 20% occur in the spine and pelvis [2]. In 80%–90% of cases, the patients already have systemic spreading at the time of diagnosis, and pulmonary metastases are the predominant site of osteosarcoma spread and the major cause of fatal outcomes [3]; these factors have all been linked to poor prognosis in osteosarcoma patients. Unfortunately, the rate of survival has not improved in 20 years. Therefore, new therapeutic targets and strategies must be sought to inhibit the progression of osteosarcoma.

Quaking(QKI) is an RNA binding protein and has been shown to serve as a tumor suppressor gene that inhibits the development of several types of tumors, including glioblastoma, astrocytic glioma, oral squamous cell carcinoma, gastric cancer and colon cancer [4–11]. Up to now, studies of QKI in tumor metastasis have been rare. Some studies have shown that QKI might inhibit the metastasis of colorectal cancer by inhibiting β-catenin post-transcriptionally [11–14]. However, the expression changes of QKI in osteosarcoma and the roles of QKI in osteosarcoma progression are unknown.

In the human genome, the miR-17-92 cluster is located on chromosome 13, which encodes six mature miRNAs (miR-17, miR-18a, miR-19a, miR-20a, miR-19b-1, and miR-92-1) [15]. The miR-17-92 cluster is known to regulate the expression of its target genes at the post-transcriptional level and to act as oncogenes. In previous studies, expression levels of miR-17-92 cluster members have been shown to be consistently upregulated in established OS cell lines and OS samples [16–19]. Moreover, the members of the miR-17-92 cluster were shown to accelerate cellular proliferation, invasion and migration in osteosarcoma cells. Recently, they were also found to influence osteosarcoma metastatic spread [19]. However, the relevant pathway through which the miR-17-92 cluster acts in the development and metastasis of osteosarcoma is still unclear.

In this study, for the first time, we revealed the expression and the pathological roles of QKI2 in osteosarcoma and found that all members of the miR-17-92 cluster could target the expression of QKI2 in osteosarcoma. Additionally, we determined the pathological roles of the miR-17-92 cluster/QKI2 regulatory axis in osteosarcoma progression.

## RESULTS

### Expression of the miR-17-92 cluster in osteosarcoma

**Figure 1:**
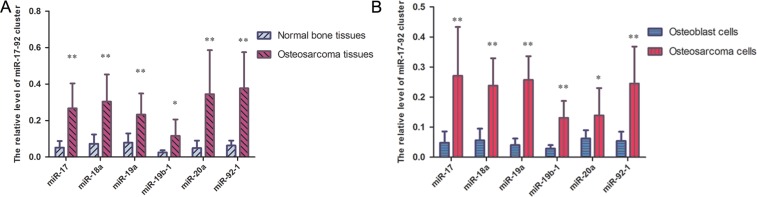
miR-17-92 cluster are highly expressed in OS samples and OS cell lines (**A**) miR-17-92 cluster are significantly increased in OS tissues compared with normal tissues, as demonstrated by RT-PCR (^*^P < 0.05, ^**^P < 0.01). (**B**) miR-17-92 cluster were higher in osteosarcoma cells than in osteoblast cells (^*^P < 0.05, ^**^P < 0.01).

In this study, the expression levels of the miR-17-92 cluster in ten pairs of osteosarcoma tissues and matched normal bone samples were quantified by quantitative real-time PCR.The expression levels of the miR-17-92 cluster, including miR-17, miR-18a, miR-19a, miR-20a, miR-19b-1 and miR-92-1, were higher in the osteosarcoma samples than those in the matched normal bone samples (Figure 1A). Similar results were also observed in the osteosarcoma cell line and the osteoblast cell line (Figure 1B). These experiments revealed that the expression levels of miR-17-92 cluster-related miRNAs are consistently upregulated in established OS cell lines and OS samples compared to controls.

### Effects of the miR-17-92 cluster on osteosarcoma cell proliferation, invasion and migration *in vitro*

**Figure 2:**
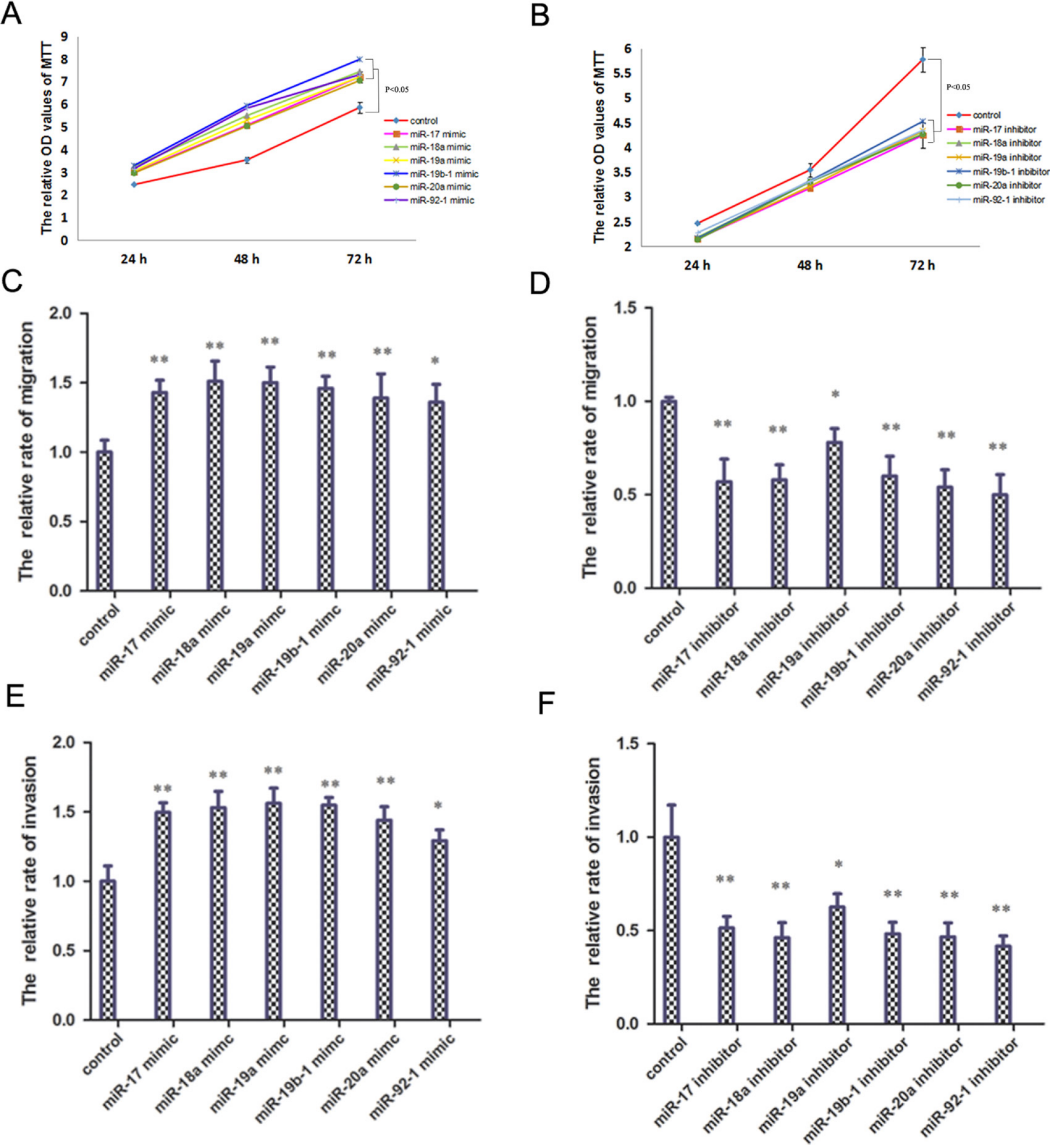
miR-17-92 cluster promote osteosarcoma cell proliferation, invasion and migration *in vitro* (**A**) Application of miR-17-92 cluster mimic promote osteosarcoma cell proliferation (*P* < 0.05). (**B**) Application of miR-17-92 cluster inhibitor reduce osteosarcoma cell proliferation (*P* < 0.05). (**C**) Application of miR-17-92 cluster mimic promote osteosarcoma cell migration (^**^P < 0.01). (**D**) Application of miR-17-92 cluster inhibitor reduce osteosarcoma cell migration (^**^P < 0.01). (**E**) Application of miR-17-92 cluster mimic promote osteosarcoma cell invasion (^**^P < 0.01). (**F**) Application of miR-17-92 cluster inhibitor reduce osteosarcoma cell invasion (^**^P < 0.01).

To determine the impact of the miR-17-92 cluster on osteosarcoma cell proliferation, invasion and migration *in vitro*, the miR-17-92 cluster mimic or inhibitor was transfected in HOS cells. The miR-17-92 cluster mimic significantly increased osteosarcoma cell proliferation as shown by cell growth assays (Figure 2A). The miR-17-92 cluster inhibitor, however, significantly reduced osteosarcoma cell proliferation (Figure 2B). The cell migration and invasion assays indicated that the miR-17-92 cluster mimic significantly promoted the migration and invasion of osteosarcoma cells (Figure 2C and 2E). Whereas the miR-17-92 cluster inhibitor significantly inhibited osteosarcoma cell migration and invasion abilities (Figure 2D and 2F). The cell growth, migration and invasion assays emphasize that the miR-17-92 cluster acts as oncogenes and promotes proliferation, migration and metastasis in osteosarcoma cells.

### The miR-17-92 cluster down-regulates QKI2 protein expression at the post-transcriptional level in osteosarcoma

**Figure 3:**
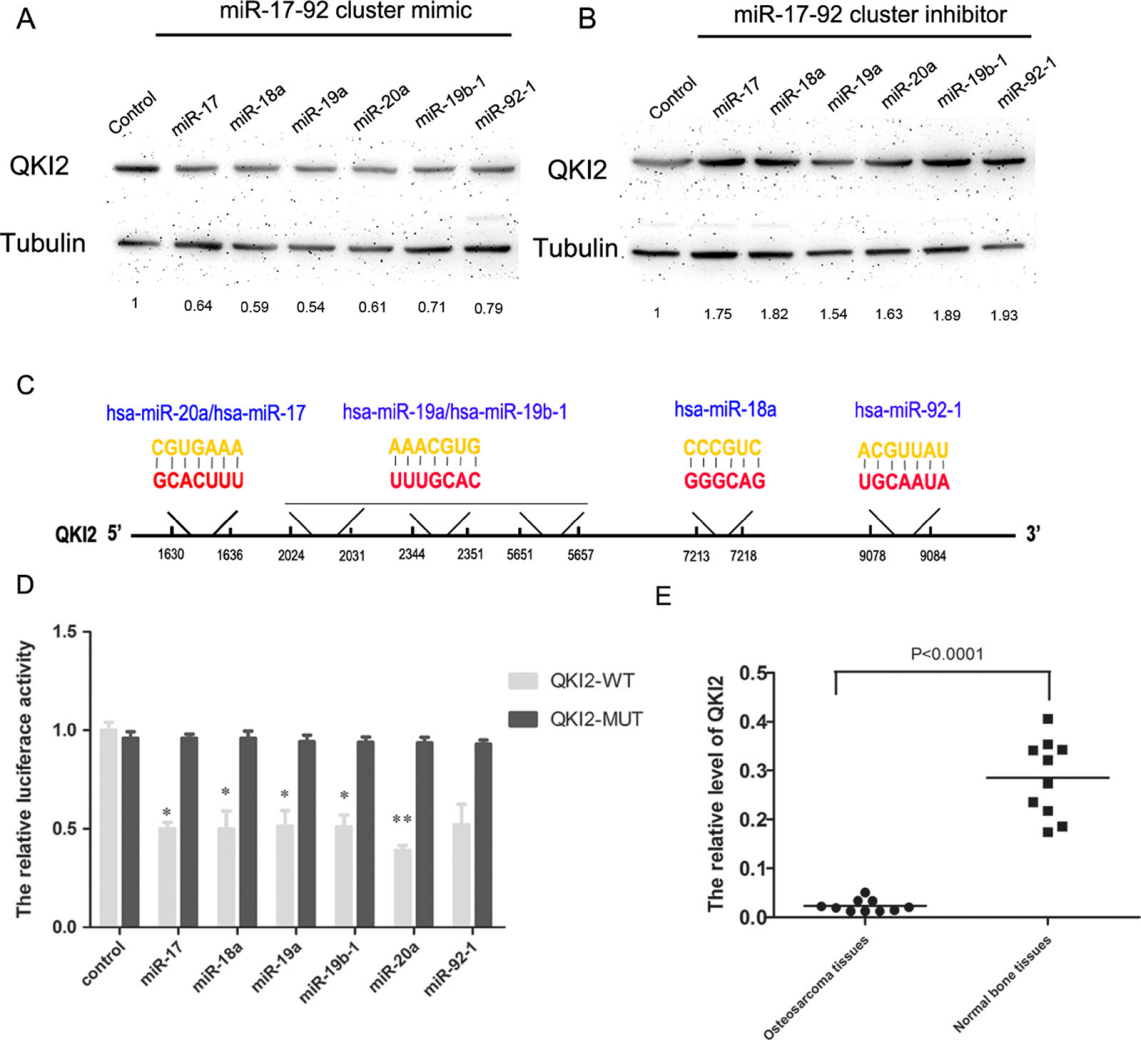
QKI2 is a target of miR-17-92 cluster and miR-17-92 cluster represses QKI2 expression in OS cells (**A**) miR-17-92 cluster mimic decreases QKI2 levels, as demonstrated via western blot analysis. (**B**) miR-17-92 cluster inhibitor increases QKI2 levels. (**C**) Diagram represents miR-17-92 with the QKI2 binding sites indicated. (**D**) miR-17-92 cluster reduces the luciferase activity of a QKI2-WT reporter plasmid, as demonstrated via a luciferase reporter assay (^*^P < 0.05, ^**^P < 0.01). (**E**) QKI2 is significantly decreased in OS tissues compared with normal tissues, as demonstrated by RT-PCR (*P* < 0.01).

In this study, we screened target genes regulated by the miR-17-92 cluster by using the online databases miRanda (http://www.microRNA.org) and TargetScan (http://www.TargetScan.org). We discovered that QKI2 might interact with the miR-17-92 cluster. To determine the relationship between QKI2 and the miR-17-92 cluster in osteosarcoma, Western blot experiments were used to analyze the expression levels of QKI2 protein in osteosarcoma cells transfected with the miR-17-92 cluster mimic or inhibitor. The miR-17-92 cluster mimic significantly decreased QKI2 expression levels in osteosarcoma cells compared with those in cells receiving the control treatment (Figure 3A). However, the miR-17-92 cluster inhibitor increased QKI2 expression (Figure 3B). To conclusively exam whether QKI2 is a direct target of the miR-17-92 cluster, a luciferase reporter assay was performed, and luciferase reporter plasmids carrying the WT or MUT form of the QKI2 3′-UTR were also constructed. Luciferase activity was measured at 24 h after co-transfection of luciferase reporter plasmids carrying the miR-17-92 cluster into HEK-293TN cells. The results demonstrated that the miR-17-92 cluster reduced the luciferase activity of only the WT QKI2 3′-UTR (Figure 3C and 3D, P < 0.01). qRT-PCR experiments were used to analyze the expression level of QKI2 protein in ten pairs of osteosarcoma and matched normal bone samples. Compared with normal bone samples, osteosarcoma tissues exhibited significantly reduced QKI2 expression levels (Figure 3E, *P* < 0.01). These results indicate that the miR-17-92 cluster down-regulates QKI2 expression after binding to QKI2 in osteosarcoma cells. Additionally, QKI2 expression is significantly down-regulated in osteosarcoma tissues.

### Effects of QKI2 on osteosarcoma cell proliferation, invasion and migration *in vitro*

**Figure 4:**
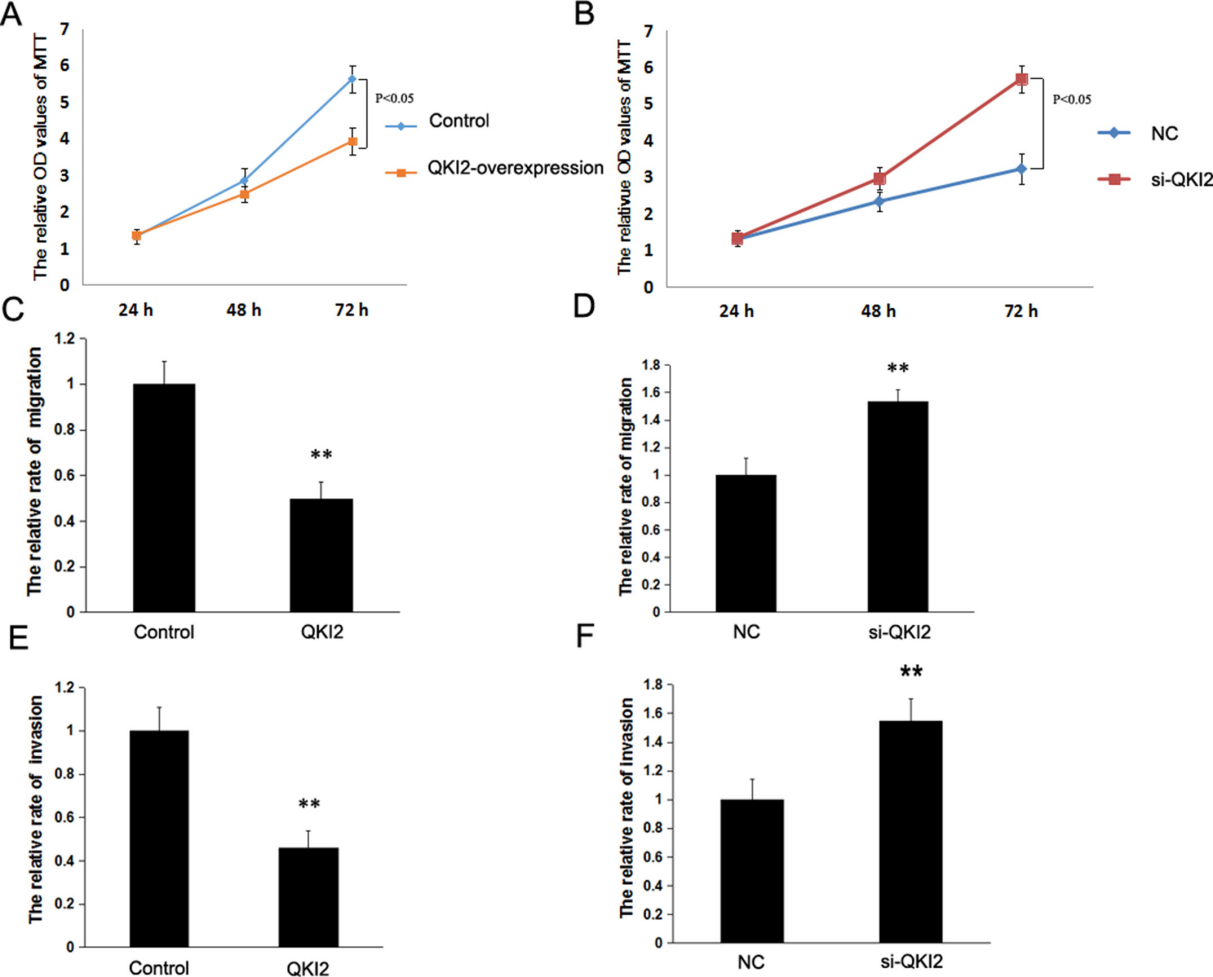
QKI2 inhibits osteosarcoma cell proliferation, invasion and migration *in vitro* (**A**) QKI2 overexpression inhibits osteosarcoma cell proliferation (*P* < 0.05). (**B**) QKI2 knockdown promotes osteosarcoma cell proliferation (*P* < 0.05). (**C**) QKI2 overexpression inhibits osteosarcoma cell migration (^**^P < 0.01). (**D**) QKI2 knockdown promotes osteosarcoma cell migration (^**^P < 0.01). (**E**) QKI2 overexpression inhibits osteosarcoma cell invasion (^**^P < 0.01). (**F**) QKI2 knockdown promotes osteosarcoma cell invasion (^**^P < 0.01).

To determine the effects of QKI2 on osteosarcoma cell proliferation, migration and invasion *in vitro*, lentiviral vectors were employed to overexpress QKI2 in HOS cells. Cell growth assays and cell migration and invasion assays demonstrated that QKI2 overexpression in osteosarcoma cells inhibited cell proliferation, migration and invasion compared with those in the control treatment (Figure 4A, 4C and 4E, *P* < 0.05), whereas QKI2 knockdown significantly promoted osteosarcoma cell proliferation, migration and invasion (Figure 4B, 4D, and 4F, *P* < 0.05).

### QKI2 protein down-regulates β-catenin protein expression by targeting β-catenin in osteosarcoma

**Figure 5:**
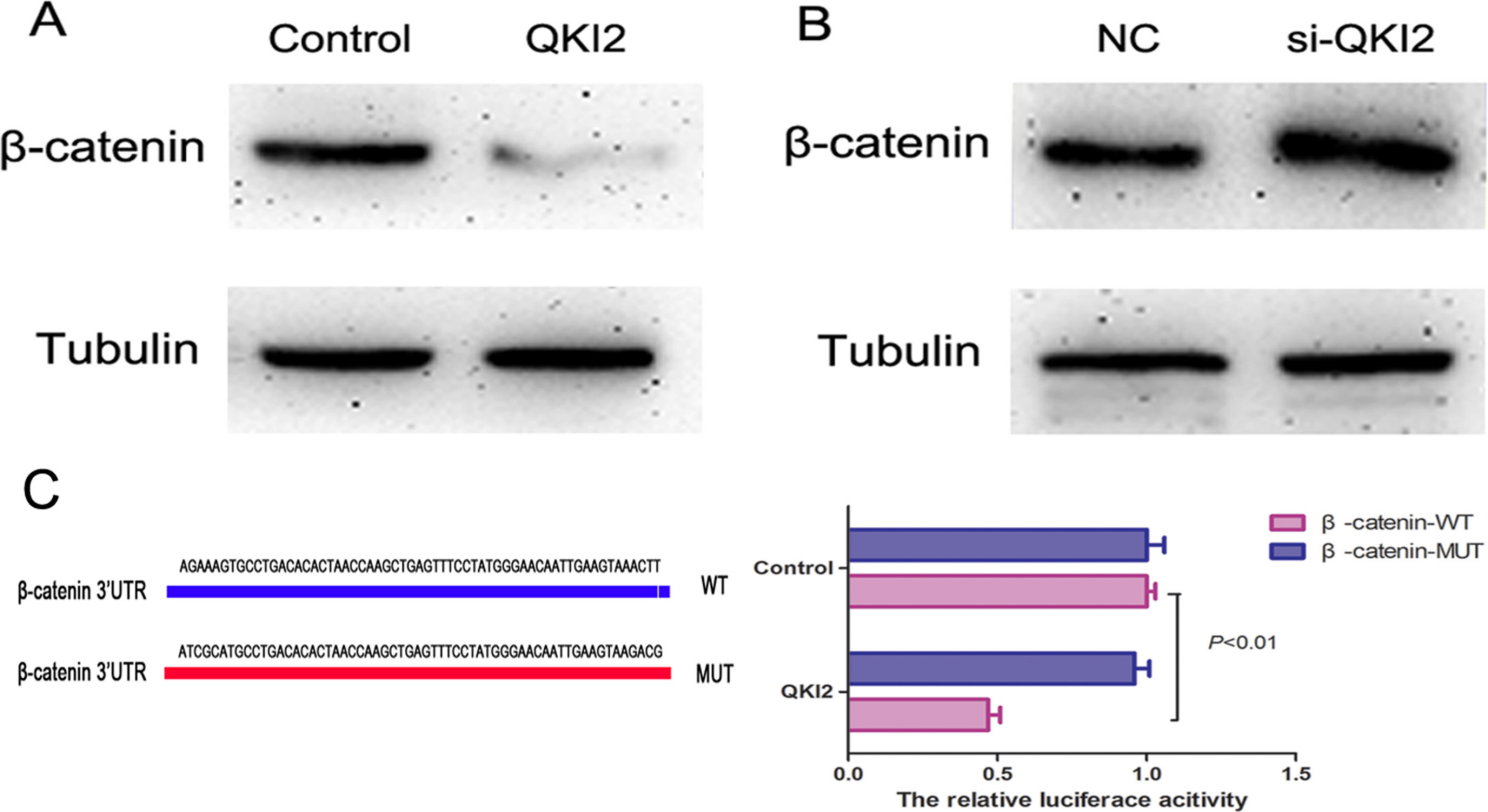
QKI2 decreases β-catenin expression in OS cells (**A**) QKI2 overexpression decreases β-catenin expression. (**B**) QKI2 knockdown promotes β-catenin expression. (**C**) QKI2 reduces the luciferase activity of a β-catenin-WT reporter plasmid (*P* < 0.01).

To determine whether QKI2 suppresses osteosarcoma cell proliferation, migration and invasion through the miR-17-92 cluster/β-catenin pathway, Western blots were used to detect β-catenin expression after QKI2 overexpression or knockdown in HOS cells. As shown in Figure 5A, QKI2 overexpression decreased β-catenin expression, whereas QKI2 knockdown increased β-catenin expression in HOS cells (Figure 5B). Furthermore, a luciferase reporter assay was also performed to estimate the relation between QKI2 and β-catenin. The luciferase reporter plasmids carrying the WT or MUT form of the β-catenin 3′-UTR were constructed. After co-transfection of luciferase reporter plasmids carrying QKI2 into HEK-293TN cells for 24 h, luciferase activity was measured. The results indicate that QKI2 inhibited the luciferase activity of only the WT β-catenin 3′-UTR (Figure 5C, *P* < 0.01). These results reveal that QKI2 competitively binds to β-catenin and down-regulates its expression in osteosarcoma cells.

## DISCUSSION

It has been shown that miRNAs can post-transcriptionally regulate the expression of protein-encoding genes through either binding to the 3’-UTR of target mRNA to partly suppress protein translation or binding to target mRNA with perfect complementarity to directly cleave target mRNA [20–22]. Consistently, miRNAs have been proven to be involved in the regulation of various cellular processes, including cell differentiation, proliferation, and apoptosis [23]. Increasing evidence suggests that miRNAs are seriously deregulated in various neoplasms and act as oncogenes(oncomirs) or tumor suppressor genes [24]. Subtle changes in individual miRNA expression levels can be sufficient to influence protein-encoding gene expression; therefore, optimal miRNA quality in each sample is a crucial prerequisite for miRNA expression analysis. However, osteosarcomas are generally treated with neoadjuvant chemotherapy before radical surgery, which potentially influences the miRNA quality. In our study, we thus chose FFPE biopsy samples of pretherapeutic primary osteosarcoma.

Some studies have shown that some members of the miR-17-92 cluster were not only overexpressed in B cell lymphoma, acute lymphoblastic leukemia, myeloid leukemia, chronic myeloid leukemia, and other hematological malignancies but also upregulated in solid tumors, such as lung cancer, gastric cancer, endometrial carcinoma, cervical carcinoma and ovarian cancer [25–30]. Thus, it is considered that members of the miR-17-92 cluster are oncomirs and contribute to promote tumor development, induce angiogenesis and inhibit apoptosis [31]. One study showed that overexpression of miR-17-5p inhibited PTEN expression in CRC and promoted cell invasion [32]. Luo Z et al. reported that miR-18a inversely regulated Dicer1 by targeting the 3’-UTR of Dicer1 and promoted the proliferation, migration and invasion of nasopharyngeal carcinoma cells by inhibiting Dicer1 expression in *in vitro* and *in vivo* experiments [33]. miR-20a is greatly upregulated in glioma stem cells and potently enhances glioma stem cell invasion through directly targeting TIMP-2 [34]. miR-19a/b, members of the miR-17-92 cluster, promote gastric cancer cell migration, invasion and metastasis by targeting MXD1, which acts as an antagonist of the c-Myc oncoprotein [35]. miR-17-5p and miR-18a, members of the miR-17-92 cluster, are up-regulated in investigated osteosarcoma cell lines [16]. In a recent study, high expression levels of the miR-17-92 cluster, including miR-19a, miR-20a, miR-19b and miR-92a, were significantly associated with prognosis and metastases in osteosarcoma patients [19]. In this study, we first reveal that the expression levels of the miR-17-92 cluster were upregulated in both OS cells and OS samples. Overexpression of the miR-17-92 cluster could promote osteosarcoma cell proliferation, invasion and migration *in vitro*.

QKI is an RNA binding protein and functions as a major post-transcriptional regulator of RNA metabolism [36–38]. Recent studies have revealed that QKI acts as a tumor suppressor gene, which is crucial for the tumorigenesis and progression of various malignant tumors, including glioblastoma, astrocytic glioma, oral squamous cell carcinoma, gastric cancer and colon cancer [4–11]. However, the function of QKI2 in osteosarcoma is unknown. In this work, to the best of our knowledge, we first discovered that QKI2 inhibited osteosarcoma cell proliferation, invasion and migration by directly targeting β-catenin. β-catenin is a well-known multifunctional protein that is involved in cellular proliferation, differentiation and apoptosis. β-catenin has been reported to be overexpressed in osteosarcoma and associated with osteosarcoma metastasis [39–41].

In conclusion, the miR-17-92 cluster/QKI2/β-catenin regulatory axis may play a key role in the proliferation and metastasis of osteosarcoma. Furthermore, the miR-17-92 cluster promotes cell proliferation, migration and invasion by competitively binding to QKI2, thereby upregulating β-catenin expression in osteosarcoma cells.

## MATERIALS AND METHODS

### Clinical samples

Ten pairs of pretherapeutic primary osteosarcoma biopsy samples and matched normal bone samples were obtained from the First/Second Affiliated Hospital of Harbin Medical University. The patients provided written informed consent to participate in this study, and this work was approved by the Ethics Committee of Harbin Medical University.

### Cell lines and cell culture

The human fetal osteoblast cell line (hFOB 1.19 cells), human osteosarcoma cell line (HOS and 143B) and HEK-293TN cell line were purchased from the Cell Bank of the Chinese Academy of Sciences (Shanghai, China). The cells were cultured in DMEM (Gibco, USA) supplemented with 10% FBS (Gibco, USA) and 2% penicillin-streptomycin (10 U/ml) at 37°C in a 5% CO_2_ atmosphere.

### Total RNA extraction and quantitative real-time RT-PCR

Total RNA from tissues and cell lines was extracted using Trizol reagent (Invitrogen). Total RNA was then converted to cDNA by reverse transcription using the TransScript^®^ One-Step gDNA Removal and cDNA Synthesis SuperMix Kit (Trans^®^, China). qRT-PCR was performed using an ABI 7900 RT-PCR system and the TransScript^®^ Tip Green qPCR SuperMix Kit (Trans^®^) according to the manufacturer's instructions. The primers were synthesized by GenePharma (Shanghai, China).

### Transfection

pWPXL lentiviral vectors were employed to overexpress QKI2 in osteosarcoma cells. The full-length sequence of QKI2 was amplified using reverse-transcription PCR, and it was digested and inserted into the pWPXL vector. To package QKI2-overexpressing lentiviral particles, the pWPXL plasmid carrying QKI2 was transfected into HEK-293TN cells together with the packaging plasmids (pSPAX2 and pMD.2G) for 48 h. Then, the supernatant was harvested and condensed using PEG. The miR-17-92 cluster mimic and the miR-17-92 cluster inhibitor were purchased from Invitrogen (Invitrogen, USA). These miRNAs were transfected into the cells using Lipofectamine 2000 (Invitrogen, USA).

### Cell growth assay

Human osteosarcoma cells (2 × 10^3^cells/well) were seeded in 96-well plates with 150 μL of serum medium and incubated at 37°C and 5% CO_2_. After 24 h, 48 h and 72 h, cell growth was evaluated by MTT assay. The MTT assay kit was purchased from Sigma-Aldrich. After 24 h, 48 h and 72 h of incubation, 10 μL of the MTT solution was added to the cell medium in each well; then, the 96-well plate was incubated at 37°C for 4 h. The culture medium was removed from each well, and 150 μL of DMSO was added to each well. The optical density (OD) values were measured at 570 nm for the cell growth assay.

### Migration and invasion assays

The migration and invasion assays were performed using a Transwell chamber (Coster, USA). First, 2 × 10^4^ cells in 200 μL of serum-free DMEM medium were seeded into the upper chamber. The lower chambers were filled with 800 μL of complete DMEM medium with 10% FBS. After incubation for 24 h at 37°C, the cells on the upper side of the chamber were softly scraped off with a cotton swab. The cells that invaded the lower side of the chamber were fixed with 1% crystal violet for 30 min before being counted. Pictures were taken using an Olympus inverted microscope (100×). The number of invaded cells was counted using ImageJ software.

### Western blot

Total protein was extracted from samples and quantitated using RIPA buffer and a BCA protein assay kit (Beyotime, China). Equal amounts of protein extracted from samples were resolved via SDS-PAGE and transferred to PVDF membranes (Bio-Rad). The membrane was blocked with nonfat milk dissolved in TBS (Tris-buffered saline) containing 0.1% Tween-20, followed by incubation with rabbit polyclonal anti-human β-catenin (Abcam, USA) and mouse monoclonal anti-human β-actin antibodies (Abcam). After being washed, the membrane was incubated with secondary peroxidase-conjugated goat anti-rabbit or anti-mouse antibodies for 1 h and visualized using enhanced chemiluminescence (ECL kit, Beyotime, China).

### Dual-luciferase reporter assay

For the luciferase assay, HEK 293 TN cells were seeded in 96-well plates. The wild-type (WT) and mutant (MUT) QKI2 3′-UTRs were inserted into a pmirGlo Dual-Luciferase miRNA Target Expression Vector (Promega, USA). The QKI2 3′-UTR reporter plasmid and miR-17-92 cluster were co-transfected into HEK-293TN cells. The luciferase activity of firefly and Renilla were measured via Dual-Luciferase Reporter Assay Kit (Promega, USA). All experiments were performed in triplicate and repeated three times.

### Statistical analysis

All statistical analyses were performed using SPSS 16 software (SPSS, USA). Significant differences between groups were determined by one-way ANOVA analysis. All results are reported as the mean ± SD. *P* < 0.05 or *P* < 0.01 was considered statistically significant.
